# The Integration of Assistive Technology and Virtual Reality for Assessment and Recovery of Post-coma Patients With Disorders of Consciousness: A New Hypothesis

**DOI:** 10.3389/fpsyg.2022.905811

**Published:** 2022-07-11

**Authors:** Fabrizio Stasolla, Leonarda Anna Vinci, Maria Cusano

**Affiliations:** Giustino Fortunato University of Benevento, Benevento, Italy

**Keywords:** post-coma, disorders of consciousness, assessment, rehabilitation, new technologies, acquired brain injuries

## Introduction

Multiple disabilities due to an outcome of coma combined with severe to profound disorders of consciousness may pose serious challenges to daily medical centers and rehabilitative settings. Beside specific pharmacological treatments delivered by specialized professionals, they may need diagnostic tools and rehabilitative interventions enabling those patients with an active role, constructive engagement, positive participation, independence, and self-determination (Pistoia et al., 2008; Lancioni et al., 2014b; Formisano et al., 2018; Kulyk, 2019). Thus, two basic objectives may be targeted within this framework, namely (a) the assessment and (b) the recovery of cognitive, motor, and communicative functioning (Lancioni et al., 2009a, 2011; Kirsch et al., 2017; de Tommaso et al., 2020).

Many clinical and research efforts have recently been devoted to the aforementioned critical features (i.e., evaluation and rehabilitation). With regard to the assessment, two main viewpoints may be emphasized. First of all, the existing literature is focused on determining the patient's state of functioning. That is, it is useful to identify whether patients are in a vegetative state or a more favorable diagnosis of minimally conscious state could be made (Lancioni et al., 2008a; Formisano et al., 2011; Pistoia and Sarà, 2012). Secondly, the dichotomy between the two above clinical conditions (i.e., vegetative state or and minimally conscious state) is critically discussed and any specific need to clarify the borderline between those two states requires to rectify more straightful strategies (Kim et al., 2012).

With regard to the rehabilitation, different approaches may be acknowledged. For instance, one may envisage environmental stimulation (Lancioni et al., 2014a, 2015). Otherwise, deep brain stimulation may be adopted (Lancioni et al., 2010b). Additionally, brain computer interface strategies may be implemented (Stasolla and De Pace, 2014). Those strategies rely on different theoretical background which may have clinical and practical implications on the role of the assessment and the role of the patient. The decision on whether the person is in a vegetative state or in a minimally conscious state should be considered crucial prior to the intervention and the setup for the intervention should be highly individualized to ensure the participant with a successful learning process (Lancioni et al., 2017).

The purpose of this paper is to argue on both assessment and rehabilitative strategies, to introduce the use of the technology as crucial means for the evaluation and the recovery of post-coma patients and disorders of consciousness, either due to a stroke or a traumatic brain injury, and to propose a new hypothesis of integration between assistive technology-based devices and virtual reality setups to improve clinical conditions of post-coma patients diagnosed with disorders of consciousness.

## Assessment Strategies

Basic assessment tools for identifying with certitude whether a patient is in a vegetative state or in a minimally conscious state commonly include behavioral scales, neuropsychological evaluation, neuroimaging techniques, and behavioral data based on learning setups (Ponsford et al., 2014; Kim et al., 2022; Ngadimon et al., 2022). Frequently, more than one tool and/or strategy are used for the evaluation (Lancioni et al., 2017). Behavioral scales probably represent the most adopted approach (Pistoia et al., 2013). An illustrative example is constituted by the JFK Coma Recovery Scale-Revised (Giacino et al., 2004) to determine the patient's responsiveness on communicative, sensorial, orientation, and motor levels of functioning. Procedural difficulties may arise whenever the patient does not have head/hand control in his/her behavioral repertoire consistent with the scale' s requests or is unable to understand verbal instructions (Bosco et al., 2010).

Neuropsychological procedures including event-related potentials such as P300 and/or mismatch negativity are also used to assess the responsiveness of patients with severe disorders of consciousness (Lancioni et al., 2009b, 2011). Thus, empirical evidences of P300 or mismatch negativity are usually viewed as basic signs of awareness or consciousness with meaningful implications for the recovery process (Estraneo et al., 2022; Pruvost-Robieux et al., 2022). Recent reviews (Pan et al., 2021; Aubinet et al., 2022) critically discussed the use of event-related potentials and emphasized the strengths to use multiple measures to enhance the significance of the findings (Calabrò et al., 2021).

Neuroimaging techniques (e.g., functional magnetic resonance imaging, fMRI) may be a reliable tool to identify potential capacities or skills even in cases of minimal or apparently absent responsiveness (Drayson, 2014: Kirsch et al., 2017; Corsi et al., 2020). In fact, the use of those techniques can strongly help researchers to catch relevant diagnostic outcomes (Kirsch et al., 2017). Nevertheless, their use is still difficult and the application may pose serious methodological challenges in a wide part of medical or rehabilitative settings due to the assessment of specific stimuli and/or the comprehension of verbal instructions (Schwarzbauer and Schafer, 2011). A valid alternative to the fMRI is represented by the positron emission tomography (Briand et al., 2020).

Behavioral data based on learning principles may refer to two different approaches. On one hand, based on classical paradigm they include the capacity of the patient to positively associate pairs of stimuli (Ricchi et al., 2022). On the other hand, based on the operant paradigm, they consist on the capacity of the patient to correctly match a behavioral response with an environmental consequence (Lee et al., 2021). Empirical evidences of learning between both approaches (i.e., a correct association between events or its positive achievement) may be considered as a non-reflective response and suggest a diagnosis of minimally conscious state (Lancioni et al., 2008b). Those strategies may be acknowledged as clinically relevant for those patients who have a minimal behavioral repertoire (e.g., eyelid or lip movements) and may pose practical problems with the JFK Coma Recovery Scale accordingly. Furthermore, learning principles based on the operant paradigm may be very useful to introduce a technological-aided program focused on promoting the participant's active role, constructive engagement, and positive participation (Stasolla et al., 2015).

## Rehabilitative Strategies

Intervention solutions for persons with disorders of consciousness may include different forms of environmental stimulation, deep brain stimulation, transcranial magnetic stimulation, brain computer interfaces, and learning-based programs or technological-mediated options (Lancioni et al., 2011, 2014a; Kim et al., 2012; Pistoia et al., 2013). An environmental stimulation program is typically delivered by the therapist/professional in charge of the patient. In its basic form it includes the presentation of daily stimulus events such as familiar music and/or verbal inputs during specific intervals of time. In a more sophisticated form, it may involve specific daily sessions with an intensive multi-sensorial intervention combined with a verbal and a physical guide of relevant events provided by the therapist (Lancioni et al., 2010a, 2014b). Although the basic form is less effective with regard to the improvement on the patient's level of alert and positive involvement in the context, the intensive form of environmental stimulation is more likely to have beneficial effects on the participant's level of attention and participation (Pape et al., 2015).

Transcranial magnetic stimulation and deep brain stimulation are considered common approaches within this specific framework whose implementation does not require any specific participation by the patient exposed to such strategy. Thus, while evidence-based support is available in the literature for that approach, studies suggest caution for its implementation because the effects on the patient's awareness and/or consciousness are mixed with regard to the amplitude and clinical significance (Kulyk, 2019).

Brain computer interfaces are systems devoted to measure brain activity and convert such activity into artificial outputs that restore, replace, enhance or support natural outputs of the Central Nervous System. Accordingly, such strategy is expected to modify the ongoing interactions between the Central Nervous System and its external or internal environment. Different techniques may be included to measure brain activity for brain computer interfaces. The most frequent method is represented by electrical signals detected through electrodes fixed invasively or non-invasively on the surface of the cortex or the scalp. Additionally, a metabolic measure may be recorded through fMRI (Lancioni et al., 2015).

Learning-based strategy are widely different from the aforementioned detailed strategies. In fact, that approach emphasizes the participant's active role, constructive engagement, and social interactions, mediated by the technology. That is, the strategy is largely designed to monitor the participant's behavioral repertoire and modify it through the manipulation of environmental consequences to ensure the participant with an independent access to positive stimulation (Stasolla et al., 2022). Those programs are aimed at fostering the participant's self-determination and reducing either caregivers or families' burden accordingly (Savoia et al., 2021). Recently, the Covid-19 pandemic outlined the development of the new technologies with an emphasis on virtual reality setups and telerehabilitation strategies to supervise patients remotely (Caprì et al., 2021; Momsen et al., 2022).

## Virtual Reality

Virtual reality (VR), and augmented reality (AR) setups, have currently been adopted as crucial means of new technological-aided programs in different area of public health, namely (a) assessment, (b) diagnosis, (c) recovery, rehabilitation, and wellbeing. With regard to rehabilitative programs, VR has been largely adopted to positively overcome neurological impairments including neurodevelopmental disorders, and neurodegenerative diseases (Stasolla, 2021; Stasolla et al., 2021). VR ensures persons with neurological impairments with sensory experiences, computer-mediated in artificial environments, enhancing virtual interactions similarly to the real life. AR, as part of VR, emphasizes an interaction in a physical condition, differently from the artificial context provided by VR. That is, VR usually requires the use of specific headsets, which may not be easily wearable for individuals with neurological disorders. Conversely, AR may be viewed as easier to use because it refers to I-PAD, tablets, and smartphones, which are more suitable to the real world (Bekkers et al., 2020; Held et al., 2020; Levin and Demers, 2021). Although widely used in patients with disorders of consciousness (Hinze et al., 2021; Kwok et al., 2021), to the best of our knowledge it has never been used in patients with acquired brain injuries, history of coma and post-coma outcomes, except for the contribution of Maggio et al. (2020). Although it may be considered as ethically controversial and questionable, such hypothesis undoubtedly merits to be empirically tested, eventually integrated with an assistive technology device, for both assessment and recovery goals.

## Discussion

Two ending conclusions may be putted forward on assessment strategies. First, using suitable corrections or supplements, behavioral scales may be considered as a practically significant solution to surpass the limitations of the scale and improve diagnostic accuracy accordingly. The aforementioned learning setups may be viewed as suitable issues in this regard (Lancioni et al., 2007a,b). Second, repeating a combined assessment between two or more strategies (i.e., behavioral scales, neuropsychological approach, and/or behavioral data), one may argue that the risk of individual's fluctuations and/or misdiagnosis might be profitably prevented (Pistoia and Sarà, 2012). Among new technologies, Hyun et al. (2021) proposed a virtual reality technology-based quantitative assessment method combined with an eye-tracking system to minimize misdiagnosis of a patient's eye movements, such as visual startle, visual fixation, and visual pursuit. Twenty healthy patients and five chronic patients in a vegetative state were systematically compared. Three stimuli were presented and visual responses data were recorded to identify valid and accurate responses to each stimulus. The system defined three of the chronic patients as showing visual fixation, undetectable through clinical assessment beforehand. Lech et al. (2021) proposed the term “Cyber-Eye” to include the emerging cognitive applications of eye-tracking interfaces for neuroscience research, clinical practice, and biomedical industry. The perspective paper suggested a brain computer interface to become less invasive, less dependent on brain activities, and more applicable as the Cyber-Eye technologies continue to develop.

Two ending considerations may be formulated on rehabilitative strategies. First, additional data are mandatory to accurately identify the impact of the different strategies and their reliability over the time and across patients. Second, systematic comparisons between procedures may be fundamental to determine the effects on a number of dimensions such as active role and positive participation (Lancioni et al., 2017). Kujawa et al. (2022) recently investigated the outcomes of an oculomotor training course aimed at the therapy of visual-spatial functions. Five patients with brain damage who were unable to communicate verbally or motorically, diagnosed between the vegetative state and the emergence from the minimally conscious state were enrolled. Over a 6-week period, the participants underwent to solved tasks associated with recognizing objects, size perception, color perception, perception of objects structure such as letters, detecting differences between images and assembling image components into the complete image with the use of an eye tracker. Findings evidenced the effectiveness of the oculomotor training based on a longer duration of the work with the eye-tracker to improve visual-spatial functions. Sanz et al. (2021) demonstrated clinical relevance and translational potential in both diagnosis and prognosis of post-coma patients with disorders of consciousness. Magnetic resonance imaging and high-density electroencephalography provided measurements of brain connectivity between functional networks, assessment of language functions, detection of covert consciousness, and prognostic markers of recovery. Positrons emission tomography could identify patients with preserved brain metabolism despite clinical unresponsiveness and could measure glucose consumption rates in targeted brain regions. Such techniques were considered encouraging and promising for both assessment and recovery purposes in clinical settings.

Finally, our new hypothesis of the integration between assistive technology-based devices and virtual reality setups may be interesting practical implications and may be adopted for both assessment and recovery purposes. For example, it may enable post-coma patients with an independent access to immersive virtual environments similar to real life. In this regard it may be viewed as a basic option of scaffolding (Dicé et al., 2018). Otherwise, one may argue that it may constitute a further form of constructive engagement and favorable occupation (Stasolla et al., 2014a) and/or of psychological wellbeing (Freda et al., 2019). Its implementation may be helpful for communicative purposes and/or challenging behaviors and the clinical relevance may be evaluated through social validation procedures (Lancioni et al., 2009a; Chiapparino et al., 2011; Stasolla et al., 2014b, 2017). For example, one may envisage the implementation in clinical settings of a virtual reality setup activated by an assistive technology-based device. Strategies of telerehabilitation (Zucchella et al., 2018; Raso et al., 2021) might be implemented. Moreover, cognitive and motor rehabilitation solutions may be embedded (Maggio et al., 2020; Daibert-Nido et al., 2021).

Future research perspectives within this framework should deal with the following topics: (a) an extension to new technological solutions combined to virtual reality-based setups to investigate the assessment and rehabilitation purposes of post-coma patients with disorders of consciousness, (b) differentiate between traumatic brain injuries, stroke, and viral causes of the coma, (c) integrate a multi-componential approach which should include behavioral scales with neuropsychological strategies, electrophysiological measures (e.g., event-related potentials), and behavioral data with the mediation of assistive technology-based devices and virtual reality setups to enhance cognitive, communicative, emotional, and motor skills of post-coma individuals with disorders of consciousness in both clinical and home-based settings.

Furthermore, the sustainability of such approach with regard to (1) its costs, (2) human resources, and (3) technological solutions available (e.g., mobile devices, wearable devices, computer-based options) should be investigated. Additionally, the inclusion in medical or rehabilitative centers should be exhaustively addressed. For instance, Bhattacharya and Pradana (in press) evaluated the literacy process in a three-year old child with Rett syndrome and significant disabilities. Two different modalities were considered, namely (a) corporal, and (b) oral. It would probably be interesting to transfer such approach to post-coma individuals with extensive motor disabilities and lack of speech.

## Author Contributions

FS conceived and wrote the paper. LV and MC edited and revised the manuscript. All authors made a substantial contribution to the article.

## Conflict of Interest

The authors declare that the research was conducted in the absence of any commercial or financial relationships that could be construed as a potential conflict of interest.

## Publisher's Note

All claims expressed in this article are solely those of the authors and do not necessarily represent those of their affiliated organizations, or those of the publisher, the editors and the reviewers. Any product that may be evaluated in this article, or claim that may be made by its manufacturer, is not guaranteed or endorsed by the publisher.
